# Intrascleral Tunnel Clamping of Fluocinolone Acetonide Implant: A Novel Scleral Fixation Technique

**DOI:** 10.3390/ph18060849

**Published:** 2025-06-06

**Authors:** Lucas Sejournet, Laurent Kodjikian, Thibaud Mathis, Alban Comet, Pierre Gascon, Frederic Matonti

**Affiliations:** 1 Department of Ophthalmology, Hôpital de la Croix-Rousse, Hospices Civils de Lyon, 69004 Lyon, France; 2 Laboratoire MATEIS, UMR-CNRS 5510, INSA, Université Claude Bernard Lyon 1, 69100 Villeurbanne, France; 3 Centre Monticelli Paradis, 13000 Marseille, France; 4 Institut de Neurosciences de La Timone, Université d’Aix-Marseille, 13000 Marseille, France

**Keywords:** fluocinolone acetonide implant, scleral fixation, macular edema

## Abstract

**Purpose:** This retrospective observational study evaluates the efficacy and safety of a novel scleral fixation technique of the fluocinolone acetonide (FAc) implant in four consecutive patients with post-surgical macular edema (PSME). **Case Presentation:** Four patients with PSME underwent intrascleral tunnel clamping (ITC) of the FAc implant due to lens defects. A 25-gauge sclerotomy was made 3.5 mm from the limbus and the implant was inserted into it until its end reached the edge of the sclera. Then, an 8-0 absorbable suture was passed through the sclera without penetrating the implant, thereby clamping the sclera around the FAc. All the patients showed improvements in best-corrected visual acuity (from a mean of 20/100 at baseline to 20/40) and central retinal thickness (from a mean of 534 µm at baseline to 318 µm) and with no recurrence of macular edema in most cases, without the need for further treatment. In addition, no anterior migration of the FAc implant or ocular hypertension was observed. This procedure effectively reduced the therapeutic burden for these patients. Although scleral fixation of the FAc implant has been described in small series of patients with successful results, this approach remains off-label. **Conclusions:** Although off-label, ITC of the FAc implant may offer a promising treatment option for patients who would otherwise remain untreated.

## 1. Introduction

Post-surgical macular edema (PMSE) is a common complication following various types of intraocular surgery, typically peaking around 6 weeks after surgery [1]. This condition can lead to significant visual impairment; however, it can often be effectively managed with topical steroid and non-steroidal anti-inflammatory drug (NSAID) eye drops [2,3]. In cases that do not respond to the initial treatment, subconjunctival injections or intravitreal injections (IVIs) of steroids may be considered [4].

However, intensive IVI regimens can lead to non-compliance and treatment failure. To overcome this situation, sustained-release drugs for intravitreal injections were developed, such as the fluocinolone acetonide (FAc) implant (Iluvien^®^, Alimera Sciences, Alpharetta, GA, USA) [5,6]. The FAc implant is a 3.5 mm × 0.3 mm non-biodegradable polymer tube that is injected into the vitreous cavity and designed to release FAc sustainably over a period of up to 3 years. Previous studies have demonstrated the safety and efficacy of the FAc implant in PSME [7,8]. Anterior migration of the corticosteroid implant in the presence of a lens defect has also been reported with both the dexamethasone implant [9,10] and FAc implant [11] with a high risk of corneal decompensation. However, the optimal treatment approach for patients with recalcitrant PMSE who do not respond adequately to topical or subconjunctival therapies remains unclear despite intravitreal corticosteroids being a well-established treatment option.

To date, this procedure has been successfully described in several case series [12,13,14] and in one interventional clinical trial [15]. A total of 15 patients with diabetic macular edema (ME) or PMSE have benefited from this procedure, with favorable anatomical and functional results. The Fluocinolone-Loop-Anchoring Technique (FLAT) involves suturing with a non-absorbable thread to suspend the implant in the vitreous cavity. However, this technique relies on the long-term stability of the suture and does not allow direct access to the implant, which could cause technical problems.

In this retrospective observational report, we describe four cases of patients with lens defects who underwent fixation of the FAc implant using a novel intrascleral tunnel clamping (ITC) technique. A 25-gauge sclerotomy was made 3.5 mm from the limbus and the implant was inserted into it until its end reached the edge of the sclera. Then, an 8-0 absorbable suture was passed through the sclera without penetrating the implant, thereby clamping the sclera around the FAc. In all the cases, the patients’ ME was either refractory or recurrent despite receiving the standard treatment for PMSE. This was mainly due to a history of multiple prior ocular surgeries, intraocular lens (IOL) dislocation, or cataract surgery complicated by posterior capsular rupture.

The Ethics Committee of the French Society of Ophthalmology approved the study conduct (IRB 00008855 Société Française d’Ophtalmologie IRB 1).

## 2. Case Presentation

### 2.1. Surgical Technique

**Figure 1:**
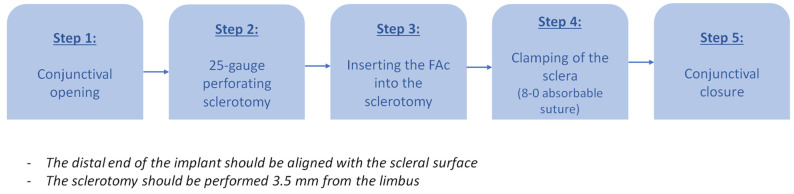
Step-by-step diagram of ITC of the FAc implant (FAc: fluocinolone acetonide; ITC: intrascleral tunnel clamping).

**Figure 2:**
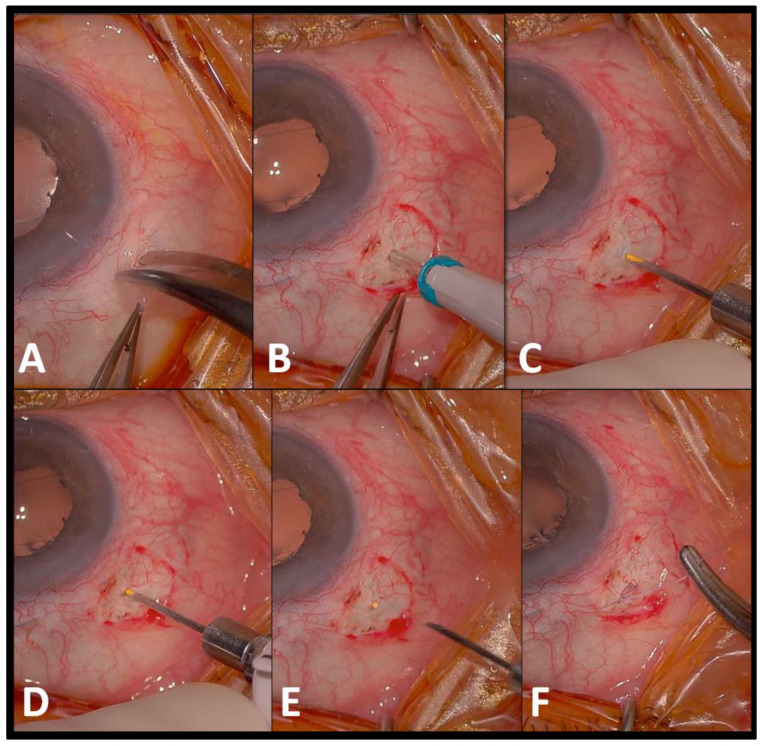
Step-by-step surgical technique for scleral fixation of the fluocinolone acetonide implant (Case 1) ((**A**) conjunctival opening; (**B**) 25-gauge sclerotomy performed 3.5 mm from the limbus; (**C**) the implant is prepared by advancing it to the tip of the injector; (**D**) insert the implant through the sclerotomy; (**E**) align the distal end of the implant with the scleral surface; (**F**) clamp the sclera using an 8-0 absorbable suture and then close the conjunctiva).

First, the conjunctiva is opened using Bonn forceps and curved Castro scissors. A perforating 25-gauge sclerotomy is made at a 90° angle, 3.5 mm posterior to the scleral-corneal limbus. The FAc implant is then inserted into the sclerotomy until its distal end aligns with the scleral edge. To secure the implant, an 8-0 absorbable suture is passed through the sclera, at 50% of its depth, as close as possible to the implant without penetrating it, effectively clamping the sclera and stabilizing the implant. Finally, the conjunctiva is repositioned over the sclerotomy (shown in Figure 1 and Figure 2). The entire procedure takes less than five minutes in all cases.

The clinical characteristics of the four cases are summarized in Table 1.

### 2.2. Case 1

A 79-year-old woman was referred to our center for management of PSME in the left eye. Her ocular history included cataract surgery complicated by posterior capsular rupture followed by sulcus IOL implantation. Four months after surgery, she developed a rhegmatogenous retinal detachment, which was treated with vitrectomy, cryotherapy, and gas tamponade. One month after the retinal detachment surgery, she developed PSME, which did not respond to steroid and NSAID eye drops.

**Figure 3:**
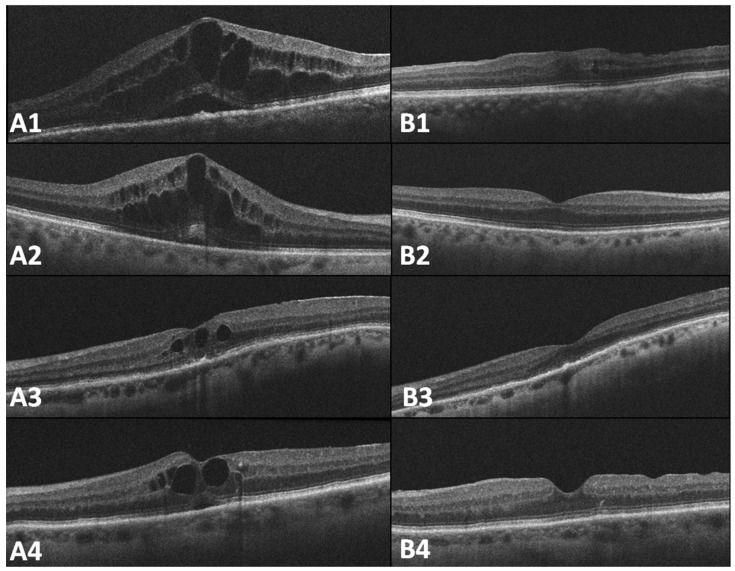
Pre- (**A1**–**A4**) and postoperative (**B1**–**B4**) macular OCT scans of cases 1, 2, 3, and 4 (OCT: optical coherence tomography).

At baseline, the best corrected visual acuity (BCVA) was 20/200 and the patient reported metamorphopsia. A slit lamp examination showed the IOL in the sulcus and vitreous in the anterior chamber. There was no contact between the IOL and the surrounding tissue, such as the iris or ciliary body. The fundus examination was unremarkable. Optical coherence tomography (OCT) showed intraretinal and subretinal fluid with a central retinal thickness (CRT) of 686 µm, as well as an epiretinal membrane (shown in Figure 3(A1)).

A 25-gauge anterior and posterior vitrectomy with epiretinal membrane peeling was performed. At the 30-day follow-up, the BCVA improved to 20/25, and the OCT showed complete resolution of ME with continued topical steroids and NSAIDs. However, at the 60-day follow-up, the BCVA had decreased to 20/40, and the OCT showed recurrence of ME. The topical treatment remained unchanged, and an additional IVI of Dex-i was administered.

Two months later, the BCVA improved to 20/32, and the OCT showed complete resolution of ME. However, due to the systematic recurrence of ME, further IVIs of Dex-i were planned. Three consecutive IVIs of Dex-i were performed every four months. Fortunately, there was no anterior chamber migration of the implant.

Given the persistent recurrence of ME despite multiple treatments, FAc implantation was planned. At the 30-day follow-up, the FAc implant had migrated into the anterior chamber, causing corneal edema and reducing the BCVA to 20/50, although the ME had resolved. The FAc implant was explanted and a new implant secured by scleral fixation using the ITC technique.

**Figure 4:**
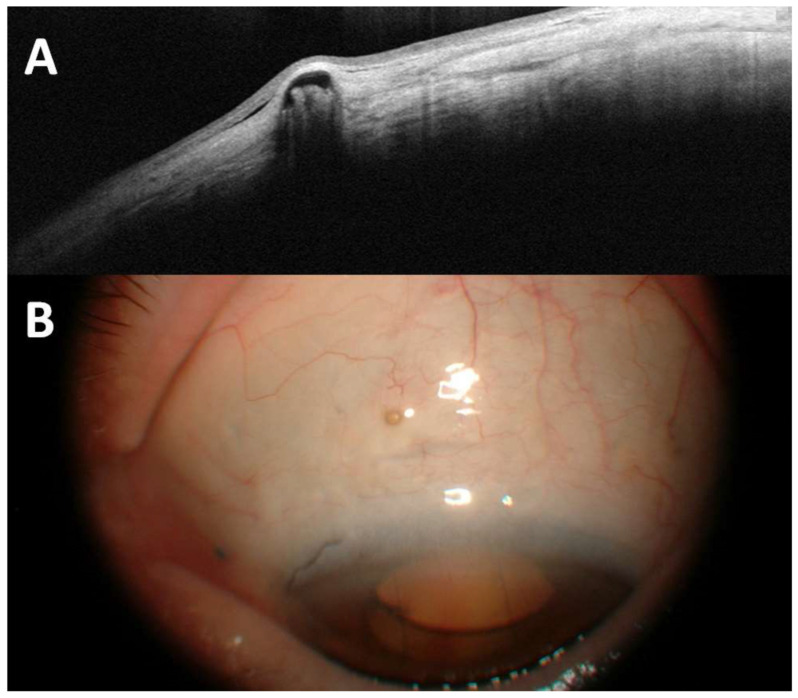
Postoperative examination of a scleral fixation of an FAc implant: (**A**) anterior segment OCT; (**B**) anterior segment photography. (FAc: fluocinolone acetonide; OCT: optical coherence tomography).

At the one-month follow-up, the BCVA had improved to 20/32, with reduced corneal edema and no recurrence of ME (CRT = 383 µm, shown in Figure 3(B1)). Eleven months later, the FAc implant was still in place (shown in Figure 4), the BCVA remained at 20/32, and there was no recurrence of ME. The patient did not require any further topical treatment or IVIs, and no ocular hypertension (OHT) was observed during the follow-up period.

### 2.3. Case 2

An 85-year-old diabetic patient was referred four months after a cataract surgery complicated by a posterior capsular rupture in the right eye. No IOL implantation was performed by the time of the surgery. The patient had a history of diabetic ME, which had been successfully treated with IVIs of Dex-i.

At baseline, the BCVA was 20/63, with no vitreous in the anterior chamber. The fundus examination was unremarkable. The OCT revealed intraretinal fluid with a CRT of 780 µm (shown in Figure 3(A2)). A 25-gauge vitrectomy was performed, with secondary IOL implantation and ITC of the FAc implant.

At the 30-day follow-up, the slit lamp examination showed an IOL in the sulcus and an FAc implant in the vitreous cavity. There was no contact between the IOL and the surrounding tissue. The OCT showed a decrease in the ME. The BCVA was 20/40. At the 90-day follow-up, the BCVA improved to 20/32.

Nine months after the surgery, the BCVA remained stable at 20/32, with no recurrence of ME (CRT = 336 µm, shown in Figure 3(B2)). The IOL and FAc implant remained in the correct position. There was no evidence of OHT during the follow-up.

### 2.4. Case 3

An 81-year-old woman was referred for spontaneous luxation of the IOL in the left eye, which occurred two years after cataract surgery.

At baseline, the BCVA was 20/100. The slit lamp examination confirmed IOL dislocation. The OCT did not show any ME. A 25-gauge vitrectomy was performed with explantation and reimplantation using the Yamane technique.

Two weeks after the procedure, the patient developed an intravitreal hemorrhage, which resolved spontaneously within a month. At this point, the BCVA had improved to 20/63, and the IOL was stable in the sulcus, without any contact with the surrounding tissue. The OCT showed no evidence of ME.

At the 90-day follow-up, the BCVA had decreased to 20/100. The slit lamp examination showed contact between the IOL and the iris, with iris atrophy and hyphemia in the anterior chamber. The OCT showed the presence of ME with a CRT of 322 µm (shown in Figure 3(A3)). A 25-gauge vitrectomy was planned, and the IOL was replaced using Prolene 10-0 sutures, and additional ITC of the FAc implant was performed.

At the 30-day follow-up, the BCVA had improved to 20/63, and the ME had completely resolved on the OCT. There was no contact between the IOL and the iris. Twelve months after the surgery, there was no ME recurrence (CRT = 247 µm, shown in Figure 3(B3)), the IOL and FAc implant were well positioned, and the BCVA remained stable at 20/63. No OHT was observed during the follow-up.

### 2.5. Case 4

An 86-year-old patient was referred to our center due to spontaneous subluxation of the IOL in the left eye. The cataract surgery performed two years prior was uneventful, and the BCVA remained stable at 20/25 without any complaints from the patient. Despite evidence of contact between the IOL and the iris, no surgical intervention was planned at that time.

Six months later, the BCVA decreased to 20/40, and the OCT showed ME with a CRT of 350 µm (shown in Figure 3(A4)) and the presence of an epiretinal membrane. Topical treatment with steroids and NSAIDs was initiated for three months. This therapy was effective, but relapse occurred each time it was discontinued. Consequently, a 25-gauge vitrectomy was performed, involving peeling of the epiretinal membrane, IOL replacement, and ITC of FAc implant. Due to the risk of anterior chamber migration, an IVI of Dex-i was not administered.

At the 30-day follow-up, the BCVA had improved to 20/32. The IOL and FAc implant were stable, and the OCT showed complete resolution of ME (CRT = 307 µm, shown in Figure 3(B4)).

At the 6-month follow-up, the BCVA remained stable at 20/25 with no recurrence of ME. Both the IOL and FAc implant remained in a stable position, without any contact with the surrounding tissue. No OHT was observed during the follow-up.

## 3. Discussion

The management of PSME in patients with aphakia or lens-diaphragm defects can be challenging due to the limited therapeutic options. It is well known that complicated cataract surgery, especially with posterior capsule rupture, increases the risk of developing PSME, making these patients particularly vulnerable. IOL implantation in the sulcus can also lead to intraocular inflammation [16]. If the IOL is in contact with the surrounding tissues, replacing the IOL is necessary to eliminate potential causes of ME. In our series, it is reasonable to assume that such contact may have contributed to the persistence of ME.

All the patients showed improvement in terms of BVCA (from a mean of 20/100 at baseline to 20/40) and CRT (from a mean of 534 µm at baseline to 318 µm). In most cases, there was no recurrence of ME without further treatment.

In our series, we did not observe any cases of OHT or anterior migration of the scleral-fixated FAc implant. In addition, we demonstrated anatomical and functional improvement in all the patients, with no recurrence of ME or need for additional treatment for six to twelve months. Although the short duration of this study raises questions about the long-term effects in these patients, previous studies have shown that the beneficial effects of FAc implants can last up to 24 months [15], which aligns with the expected duration of action for the FAc implant.

The FLAT technique has previously been described to treat these patients [14]. However, this procedure relies on the stability of a non-absorbable suture and does not provide direct access to the FAc implant. In contrast, the ITC technique appears to be faster and easier to perform. In addition, the direct subconjunctival access allows for straightforward removal in the event of complications such as OHT or replacement when the efficacy of the implant fades. The technique also appears to be safe, with no reported cases of implant dislocation or conjunctival erosion.

Although scleral fixation of Dex-i has been described previously [17], it was not used in our patients. The shorter duration of action of Dex-i and the risk of implant biodegradation, which could lead to anterior migration and cause severe corneal complications, led us to opt for the FAc implant instead [18].

Although this procedure has shown encouraging results in a small cohort of patients, concerns remain regarding the potential loss of efficacy of the FAc implant over time. Repeated intraocular surgery to replace the implant each time its efficacy diminishes may be an overly invasive approach for patients with chronic ME.

A major limitation of our study is the small sample size, which limits the generalizability of our results. Nevertheless, this procedure is rarely described in the literature, with only 15 cases reported to date. Therefore, further research and larger case series are necessary to evaluate the long-term safety and potential efficacy of this technique before it can be considered for wider use.

## 4. Conclusions

For patients with PSME and aphakia or lens-diaphragm defects where other therapeutic options are limited, scleral fixation of the FAc implant may be a viable treatment option. Although the number of reported cases in the literature is still small, the procedure has shown encouraging results. This novel ITC technique provides easier and quicker access to the implant when needed, which could improve the management of these complex cases.

Due to the small sample size of this report, it is not currently possible to draw any conclusions regarding the generalizability of these results. Further studies involving larger patient cohorts are required to improve our understanding of the long-term outcomes, particularly with regard to the potential loss of FAc implant efficacy after two to three years.

## Figures and Tables

**Table 1:** Clinical characteristics of the patients at baseline and after ITC of FAc implant (FAc: fluocinolone acetonide; ITC: intrascleral tunnel clamping; CRT: central retinal thickness; F: female; M: male; IOL: intraocular lens; ERM: epiretinal membrane).

Case	Age (Years)	Sex	Surgical History	Baseline BVCA (Snellen)	Baseline CRT (µm)	Lens Status	Final BVCA (Snellen)	Final CRT (µm)	Follow-Up (Months)
1	79	F	Phacoemusification (posterior capsular rupture) Vitrectomy, cryotherapy Vitrectomy, ERM peeling	20/200	686	Sulcus IOL	20/32	383	11
2	85	M	Phacoemulsification (posterior capsular rupture)	20/63	780	Aphakia	20/32	336	9
3	81	F	Phacoemulsification Vitrectomy, Yamane	20/100	322	IOL (Yamane)	20/63	247	12
4	86	F	Phacoemulsification	20/40	350	IOL (Subluxation)	20/32	307	6

## Data Availability

Data is contained within the article.
